# Rosai-Dorfman disease: an infrequent entity with heterogeneous presentation. Case series of six patients^؆^

**DOI:** 10.1016/j.abd.2025.501216

**Published:** 2025-11-07

**Authors:** Daniela Uribe, Samuel Ibáñez, Paula Giacaman, Faustino Alonso, Kharla Pizarro, María Martin, Verónica Catalán, Matías Garate, Leonardo Peruilh, Manuel Rodríguez, Daniela Merino

**Affiliations:** a Department of Dermatology, Clinical Hospital of the University of Chile, University of Chile, Santiago, Chile; b School of Medicine, Pontificia Universidad Católica de Chile, Santiago, Chile; c Service of Dermatology, Hospital San José, Santiago, Chile; d Service of Pathologic Anatomy, Hospital San José, Santiago, Chile; e Department of Dermatology, Clínica Alemana de Santiago, Universidad del Desarrollo, Santiago, Chile

Dear Editor,

Rosai-Dorfman disease (RDD) or sinus histiocytosis with massive lymphadenopathy is a rare, benign histiocytic lymphoproliferative disorder with a prevalence of 1:200.000, most frequently in children and young adults. RDD is classified into classical or nodal (57%) and extranodal (43%) forms. The cutaneous presentation is the second most frequently affected extranodal site (10%). The cutaneous-only subtype known as cutaneous Rosai-Dorfman disease (CRDD) is uncommon (3%), does not present systemic or lymphonodal involvement, and is a diagnosis of exclusion. Histopathology is characterized by large histiocyte proliferation with pale cytoplasm, hyperchromatic nucleus, and prominent nucleolus, usually accompanied by multiple plasma cells. A key finding is emperipolesis, where intact inflammatory cells are engulfed by histiocytes, differentiating it from phagocytosis. Immunohistochemical (IHC) staining typically reveals S100+, CD68+, and CD1a- histiocytes. Less frequent markers include CD163+, CD14+, and CD207-.^1^, ^2^, ^3^

Complementary investigation includes laboratory and imaging studies. Magnetic resonance imaging (MRI) is preferred for initial staging in pediatric patients, while adults benefit from positron emission tomography ‒ computed tomography (PET-CT).^2^, ^4^ Treatment varies depending on the presence of skin or systemic involvement, with highly variable responses.^4^, ^5^

Prognosis depends on systemic/nodal involvement. Nodal or cutaneous disease can remit spontaneously in 20%‒50% of cases. The prognosis is less favorable in multisystemic disease, with mortality of 7% to 12%.^4^

The aim of this communication is to describe six cases of cutaneous RDD seen at a Chilean public hospital, highlighting its heterogeneous clinical presentation and the importance of accurate recognition for appropriate management.

## Case 1

**Figure 1:**
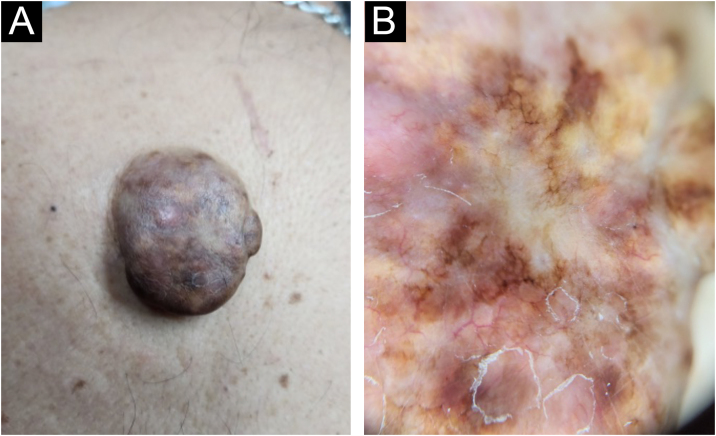
Clinical and dermatoscopic images of case 1 [extranodal cutaneous RDD]. (A) Single skin lesion on the medial dorsum. (B) Dermatoscopic image: disorganized white-yellowish areas and pigmentary network patches on a red-milky background, with telangiectatic vessels.

A 61-year-old man presented with an asymptomatic single lesion on the dorsum of four months of evolution. Examination revealed a brown exophytic tumor, with a sessile base and smooth and soft surface in the interscapular area (Fig. 1A). No other findings were detected. The dermatoscopic image of the lesion described in this case can be seen in Fig. 1-B. Systemic workup excluded extracutaneous involvement, supporting a diagnosis of CRDD.

## Case 2

**Figure 2:**
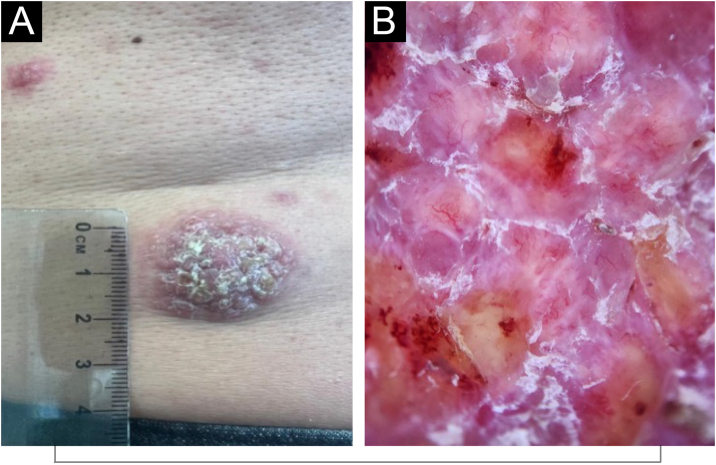
Clinical and dermatoscopic images of case 2 [systemic RDD]. (A) Lumbar lesion. (B) Dermatoscopic image: unstructured yellowish areas surrounded by milky-red regions associated with in focus arborizing vessels, whitish scales, and some intracorneal hemorrhagic crusts.

A 54-year-old man was referred for multiple lesions of four-months of evolution, associated with little pain and pruritus, night sweats, weight loss, and general malaise. On physical examination: multiple erythematous-violaceous exophytic tumors with a rough, hyperkeratotic surface and well-defined borders, of variable sizes (ranging from 10 to 40 mm), located in the bilateral lumbar region, left dorsal area, left shoulder, and right lower extremity. Bilateral firm inguinal lymphadenopathy was noted. The clinical and dermatoscopic image of the largest lesion can be seen in Fig. 2. Complementary studies confirmed nodal involvement, consistent with systemic RDD.

## Case 3

**Figure 3:**
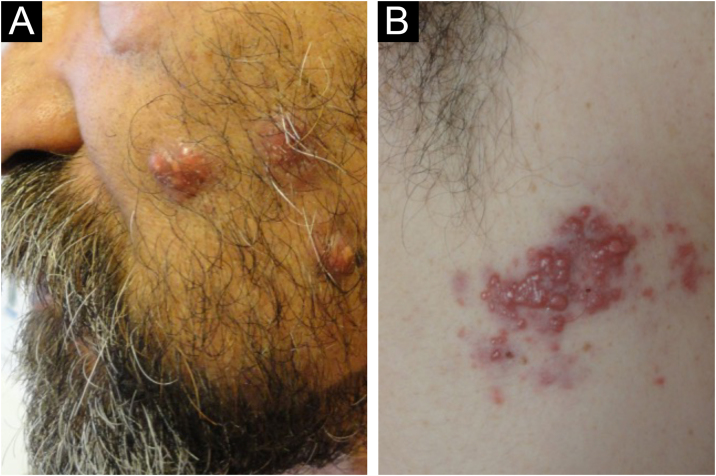
Clinical images of Case 3 [systemic RDD]. (A) Facial lesions. (B) Axillary lesions.

A 60-year-old man. Consulting by the appearance of multiple lesions three-months old, located on the face, anterior and posterior trunk. On examination: multiple rounded, erythematous-violaceous plaques, well circumscribed, irregular surface, with whitish punctiform areas. Lesions on the anterior trunk (sternal area), left cheek, and middle/lower dorsum. In addition, an irregular plaque of 30 × 20 mm, formed by multiple confluent erythematous papules of 2‒4 mm, grouped on an irregular erythematous-violaceous background, was found in the right axilla (Fig. 3). The imaging studies showed systemic involvement.

## Case 4

**Figure 4:**
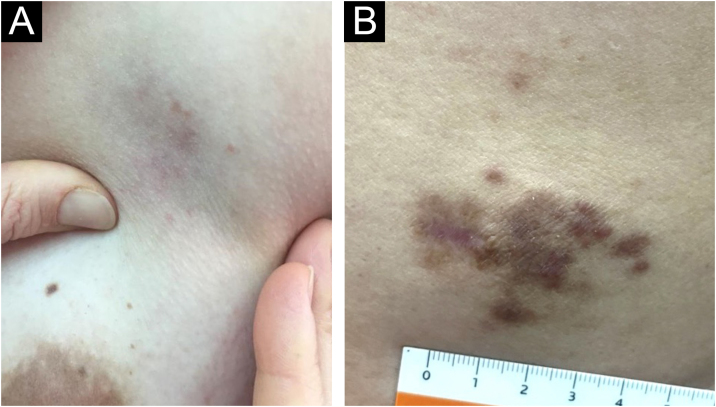
Clinical images of case 4 and 5 [Extranodal cutaneous RDD]. (A) Case 4. Single skin lesion on left breast (subcutaneous nodule evident to deep palpation). (B) Case 5. Dorsal lesion.

28-year-old woman. Referred from the breast pathology unit (BPU) due to an increase in volume of the left breast, of unspecified evolution time (at least 6-months), and whose study by BPU was negative. On physical examination: subcutaneous nodule of indurated consistency, partially mobile, approximately 40 mm, painless, evident to deep palpation. The overlying cutaneous surface showed a few faint pink-colored papules of 1‒2 mm, with a slightly erythematous-violaceous background (Fig. 4A). There were also ipsilateral axillary lymph nodes. Extension studies excluded systemic involvement, supporting the diagnosis of exclusively cutaneous disease.

## Case 5

A 48-year-old man, with a history of arterial hypertension presented with two asymptomatic skin lesions on the dorsum and abdomen, of four-months of evolution. Physical exam showed a 5 × 3 cm subcutaneous firm and poorly defined nodule on the right dorsum, with erythematous-violaceous macules grouped on the skin surface (Fig. 4B). Additionally, a subcutaneous mass of 10 × 10 cm was found in the right iliac fossa. PET-CT shows no evidence of systemic involvement, supporting the diagnosis of exclusively cutaneous disease.

## Case 6

A 34-year-old man with a history of arterial hypertension. He consulted due to multiple palpable subcutaneous nodules on the dorsum of eight-months of evolution, preceded by sudden dysphonia six-months earlier (interpreted as recurrent laryngeal nerve palsy), without findings in the neck and chest Computed Tomography (CT). In a second chest CT scan, requested after the appearance of skin lesions, a mediastinal mass compressing the path of the recurrent laryngeal nerve was observed. Cutaneous metastasis of an undetermined primary tumor was suspected.

**Figure 5:**
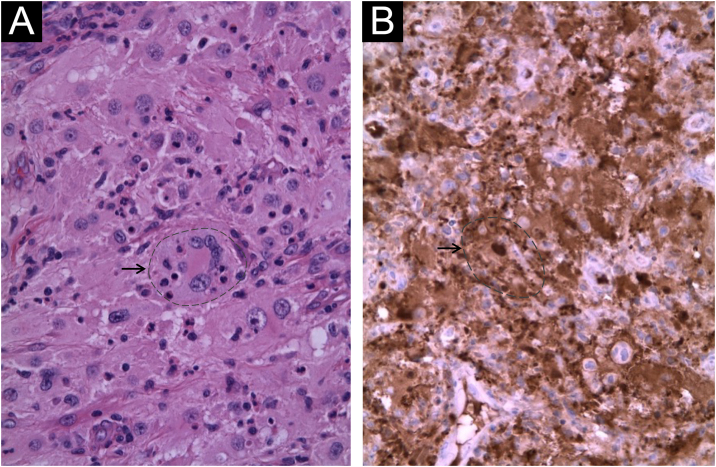
Histopathological images of case 2. (A) Hematoxylin and eosin (20×, case 2). Foamy histiocytes with pale eosinophilic cytoplasm and emperipolesis, accompanied by lymphocytes, neutrophils, plasma cells and eosinophils. (B) Immunohistochemistry (IHQ) positive for S100 in histiocytes (20×, case 2), especially notable in the central histiocyte of the image, which shows the phenomenon of emperipolesis. Emperipolesis is indicated by the arrow and the dashed line in both figures.

In the six cases described, skin biopsy was performed, and all reported a dermal histiocytosis with morphological and immunohistochemical findings compatible with RDD. Histopathology with hematoxylin and eosin (H&E) staining and immunohistochemistry (IHQ) of one of the lesions from case 2 can be observed in Fig. 5. The complementary study determined that cases 2, 3 and 6 had systemic involvement, and were referred to the hematology-oncology service, with partial response during the first months of oncology treatment. Cases 1, 4 and 5 had exclusively cutaneous involvement, which were successfully managed with a surgical approach by dermatology.

The diagnosis of RDD can be challenging, requiring a high degree of suspicion due to its rare and heterogeneous presentation, which often leads to a wide spectrum of differential diagnoses. The diagnosis is confirmed by histopathological biopsy. However, the clinical and histopathological features of the lesions alone cannot predict whether there is extracutaneous involvement. Therefore, a complementary laboratory (including complete blood count, liver function, and coagulation and urine test) and imaging studies are essential to assess systemic involvement, disease extent, and associated disorders.

This miniseries of cases aimed to highlight the diverse and atypical dermatological manifestations of an uncommon disease and thus provide tools that contribute to its proper recognition, as these manifestations may represent the initial sign of a pathology with potential systemic involvement.

## ORCID ID

Daniela Uribe: 0009-0001-2180-3924

Samuel Ibáñez: 0009-0000-8593-6171

Paula Giacaman: 0000-0002-8144-8872

Faustino Alonso: 0000-0003-1181-1039

Kharla Pizarro: 0009-0006-0894-6249

María Martin: 0009-0001-6384-0483

Verónica Catalán: 0000-0002-4624-4217

Matías Garate: 0000-0002-2814-0211

Leonardo Peruilh: 0000-0002-1803-5354

Manuel Rodríguez: 0000-0002-5385-6916

Daniela Merino: 0009-0002-5560-4170

## Financial support

None declared.

## Authors' contributions

Daniela Uribe: Approval of the final version of the manuscript; critical literature review; data collection, analysis and interpretation; effective participation in research orientation; preparation and writing of the manuscript; study conception and planning.

Samuel Ibáñez: Approval of the final version of the manuscript; critical literature review; preparation and writing of the manuscript.

Paula Giacaman: Data collection, analysis and interpretation; effective participation in research orientation; intellectual participation in propaedeutic and/or therapeutic management of studied cases; study conception and planning.

Faustino Alonso: Approval of the final version of the manuscript; intellectual participation in propaedeutic and/or therapeutic management of studied cases; manuscript critical review.

Verónica Catalán: Approval of the final version of the manuscript; intellectual participation in propaedeutic and/or therapeutic management of studied cases; manuscript critical review.

Kharla Pizarro: Data collection, analysis and interpretation; Intellectual participation in propaedeutic and/or therapeutic management of studied cases; manuscript critical review.

María Martin: Intellectual participation in propaedeutic and/or therapeutic management of studied cases.

Matías Garate: Intellectual participation in propaedeutic and/or therapeutic management of studied cases.

Leonardo Peruilh: Intellectual participation in propaedeutic and/or therapeutic management of studied cases.

Manuel Rodríguez: Critical literature review; data collection, analysis and interpretation.

Daniela Merino: Intellectual participation in propaedeutic and/or therapeutic management of studied cases.

## Research data availability

The entire dataset supporting the results of this study was published in this article.

## Conflicts of interest

None declared.
